# First-Line Tislelizumab Combined with Bevacizumab and CAPOX for Metastatic Gastroesophageal Adenocarcinoma with Low Programmed Death-Ligand 1 Expression

**DOI:** 10.34133/cancomm.0024

**Published:** 2026-04-16

**Authors:** Ru Jia, Yan-Rong Wang, Fang-Fang Liu, Yue Ma, Hai-Yan Si, Lu Han, Miao-Miao Gou, Zhao-Li Tan, Nan Zhang, Guo-Chao Deng, Meng-Jiao Fan, Yue Shi, Yao-Yue Zhang, Yu-Shan Jia, Jun-Nan Xu, Xiao-Xing Su, Quan-Li Han, Zhi-Kuan Wang, Guang-Hai Dai

**Affiliations:** ^1^ Senior Department of Oncology, Chinese PLA General Hospital, Beijing, P. R. China.; ^2^ Senior Department of Oncology, Medical School of Chinese PLA, Beijing, P. R. China.; ^3^ Kanghui Medicine, Kanghui Biotechnology Co., Ltd., Shenyang, Liaoning, P. R. China.

Gastroesophageal adenocarcinoma (GEA), including gastric, gastroesophageal junction, and esophageal adenocarcinoma, remains a highly aggressive malignancy that is often diagnosed at an advanced stage and is associated with poor prognosis worldwide. Programmed cell death protein 1 (PD-1) inhibitor plus chemotherapy has been established as a standard first-line regimen for patients with human epidermal growth factor receptor 2 (HER2)-negative advanced GEA, demonstrating a significant overall survival (OS) benefit over chemotherapy alone [1–3]. However, the magnitude of benefit varies according to programmed death-ligand 1 (PD-L1) combined positive score (CPS), with greater efficacy consistently observed in patients with higher CPS values [1,3]. Accordingly, major guidelines, including those from the National Comprehensive Cancer Network, European Society for Medical Oncology, and American Society of Clinical Oncology, preferentially recommend PD-1 inhibitors plus chemotherapy for patients with CPS ≥5 [4–6], leaving the optimal strategy for patients with CPS <5 as an important unmet need.

Primary resistance to PD-1 inhibitors is associated with an immunosuppressive tumor microenvironment, which may in part be driven by abnormal angiogenesis mediated by vascular endothelial growth factor (VEGF) and its receptor (VEGFR). Vascular dysfunction limits effector T cell infiltration and promotes immunosuppressive cell recruitment, whereas anti-VEGF/VEGFR therapy can normalize tumor vasculature and enhance antitumor immune responses, providing a rationale for combining anti-VEGF agents with PD-1 inhibitors [7]. However, in the phase III LEAP-015 trial, lenvatinib plus pembrolizumab and chemotherapy failed to achieve a statistically significant OS benefit in patients with GEA [8]. This may be partly attributable to the higher incidence of grade ≥3 toxicities associated with lenvatinib and to early discontinuation of chemotherapy in the experimental arm. Moreover, clinical evidence for combining anti-VEGF/VEGFR therapy with PD-1 inhibitors and chemotherapy in GEA remains limited.

Bevacizumab is a monoclonal anti-VEGF antibody targeting VEGF-A and has a pharmacologic profile distinct from that of small-molecule VEGFR tyrosine kinase inhibitors, including intravenous administration, a prolonged half-life, and selective VEGF blockade, which may offer an alternative approach to VEGF inhibition when combined with PD-1 inhibitors and chemotherapy [7,9,10]. On this basis, we conducted a single-arm, single-center, exploratory phase II study (NCT05299476) evaluating first-line tislelizumab (a PD-1 inhibitor) in combination with bevacizumab and capecitabine plus oxaliplatin (CAPOX) in patients with PD-L1 CPS <5. Between 2022 April 25 and 2024 December 12, a total of 32 patients with locally advanced or metastatic GEA and PD-L1 CPS <5 were enrolled (Supplementary Materials and Fig. S1). Tislelizumab (200 mg) and bevacizumab (7.5 mg/kg) were administered intravenously on day 1 of each 3-week cycle, with capecitabine (1,000 mg/m^2^ orally twice daily, days 1 to 14) and oxaliplatin (130 mg/m^2^ intravenously, day 1, for up to 8 cycles). Treatment continued until disease progression, unacceptable toxicity, withdrawal of consent, or investigator decision. The primary endpoint was the 6-month progression-free survival (PFS) rate. All patients received at least one dose of study treatment and were included in the full analysis set (FAS). Baseline characteristics are summarized in Table S1.

Within the FAS, the objective response rate was 56.2% (95% confidence interval [CI] 37.7% to 73.6%) and the disease control rate was 100.0% (95% CI 89.1% to 100.0%) (Table S2). Eighteen patients (56.2%) achieved partial response (PR), while 14 (43.8%), including 5 with nontarget lesions at the baseline, achieved stable disease (SD) (Fig. 1). Among 27 patients with measurable disease, 18 patients (66.7%) achieved PR and 9 (33.3%) achieved SD (Fig. 1). The objective response rate was 66.7% (95% CI 46.0% to 83.5%), and the disease control rate was 100.0% (95% CI 87.2% to 100.0%; Table S2). The median duration of response was 8.8 (95% CI 6.2 to 11.4) months, and the median time to response was 1.7 (95% CI 1.1 to 2.2) months (Table S2). At the data cutoff (2025 January 20), the median follow-up was 14.4 (interquartile range, 11.4 to 27.7) months. The 6-month PFS rate was 93.0% (95% CI 84.0% to 100.0%), and the median PFS was 10.0 (95% CI 6.9 to 13.0) months (Fig. 1). A total of 12 deaths (37.5%) had occurred among the 32 patients, and the median OS was 18.5 (95% CI 16.3 to 20.7) months. The 12- and 18-month OS rates were 85.7% (95% CI 73.7% to 99.7%) and 51.3% (95% CI 31.6% to 83.3%), respectively (Fig. 1). PFS appeared generally comparable between patients with PD-L1 CPS <1 (*n* = 11) and CPS 1 to 4 (*n* = 21) (hazard ratio [HR] = 1.03, 95% CI 0.41 to 2.56). Numerical differences in PFS were observed across selected baseline characteristics, including sex (males *n* = 22 vs. females *n* = 10; HR = 0.37, 95% CI 0.14 to 0.95), baseline CA19-9 level (≤27 U/ml, *n* = 23 vs. >27 U/ml, *n* = 9; HR = 0.33, 95% CI 0.11 to 0.98), and CA72-4 level (≤6.9 U/ml, *n* = 15 vs. >6.9 U/ml, *n* = 17; HR = 0.27, 95% CI 0.10 to 0.77; Fig. S2A). Numerical differences in OS were observed according to baseline CA19-9 levels (≤27 U/ml, *n* = 23 vs. >27 U/ml, *n* = 9; HR = 0.10, 95% CI 0.02 to 0.51; Fig. S2B). Twenty patients received subsequent therapies, as summarized in Table S3.

**Fig. 1.**
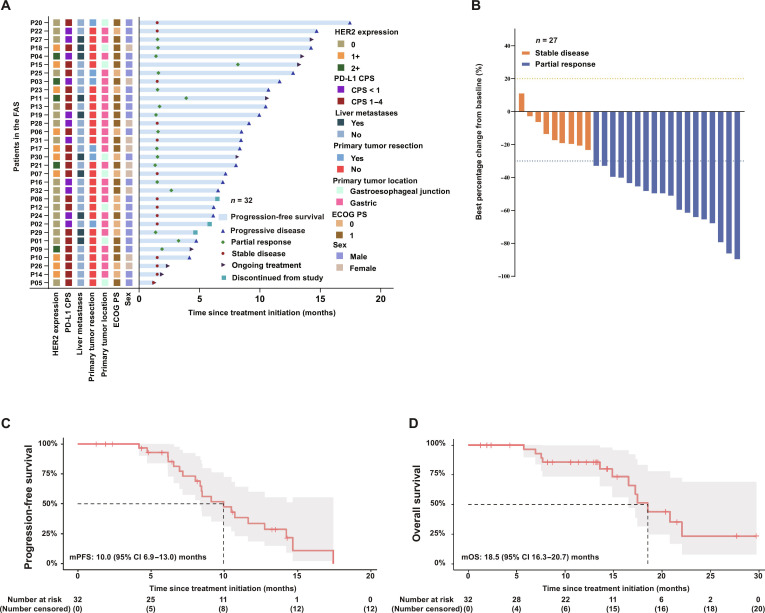
Efficacy of tislelizumab combined with bevacizumab and CAPOX chemotherapy as first-line treatment in patients with HER2-negative, PD-L1 CPS <5 locally advanced or metastatic gastroesophageal adenocarcinoma. (A) Swimmer plot showing the duration of treatment and best overall response for all patients in the FAS population (*n* = 32). (B) Waterfall plot depicting the best percentage change from baseline in the sum of target lesion diameters, as assessed by the investigator per RECIST version 1.1. The analysis includes 27 patients from the FAS who had measurable lesions at baseline. Each bar represents an individual patient. (C) Kaplan–Meier curves for PFS in the FAS population. Tick marks on the curve represent individual censored patients. (D) Kaplan–Meier curves for OS in the FAS population. Tick marks indicate censored patients. The FAS population included patients who received at least one dose of study treatment. PFS was defined as the time from treatment initiation to the first documented PD or death from any cause. OS was defined as the time from treatment initiation to death from any cause. CAPOX, capecitabine plus oxaliplatin; CI, confidence interval; CPS, combined positive score; FAS, full analysis set; HER2, human epidermal growth factor receptor 2; mPFS, median progression-free survival; mOS, median overall survival; OS, overall survival; PD, progressive disease; PD-L1, programmed death-ligand 1; PFS, progression-free survival; PR, partial response; RECIST, Response Evaluation Criteria in Solid Tumors; SD, stable disease; ECOG PS, Eastern Cooperative Oncology Group Performance Status.

Among the 12 patients with tumor tissue samples included in the exploratory biomarker analyses, 6 with PR were classified as responders, while the remaining 6 with SD were considered nonresponders. RNA sequencing revealed that nonresponders showed higher expression of keratin-6A (*KRT6A*) and repetin (*RPTN*) (keratin-associated genes) and desmoglein 3 (*DSG3*) and *DSG4* (genes related to the epithelial barrier function) (Fig. S3A). Additionally, RNA sequencing data showed higher interleukin-17A (*IL-17A*) expression in responders (Fig. S3B), and *IL-17A* expression was associated with both PFS and OS (Fig. S3C and D). Immunohistochemistry and baseline serum analyses consistently showed higher IL-17A levels in responders (Fig. S3E to G). Furthermore, multiplex immunohistochemistry demonstrated a higher infiltration of CD66b^+^ neutrophils in responders compared to that in nonresponders (Fig. S3H and I).

All 32 patients were evaluable for safety. Treatment-related adverse events (TRAEs) of any grade occurred in 27 patients (84.4%), most commonly decreased neutrophil count (59.4%), nausea (46.9%), decreased platelet count (40.6%), and anorexia (40.6%). Grade 3 to 4 TRAEs occurred in 14 patients (43.8%), mainly decreased neutrophil count (21.9%) and hand-foot syndrome (9.4%; Table S4). Immune-related adverse events were reported in 11 patients (34.4%), with grade ≥3 events in 2 patients, including grade 3 rash (*n* = 1) and grade 4 hyperbilirubinemia (*n* = 1). Bevacizumab-related adverse events included hypertension (grade 2, *n* = 1; grade 3, *n* = 1) and bleeding (all grade 2, *n* = 3) (Table S4). No treatment-related deaths occurred.

Nonetheless, this single-arm, single-center phase II study has a small sample size and limited statistical power. The absence of a comparator arm prevents definitive attribution of observed outcomes to the investigational regimen rather than potential population selection or bias. Results are hypothesis generating rather than evidence of superiority. Subgroup and biomarker analyses were conducted in small subsets and are therefore presented as exploratory and descriptive only, with no adjustment for multiple comparisons. Subgroup analyses were not sufficiently powered to support formal between-group comparisons and were not intended to support subgroup inference. Biomarker analyses lacked functional or causal validation. Consequently, findings from subgroup and biomarker analyses should be considered preliminary and require confirmation in larger, independent cohorts. Despite these limitations, this phase II trial demonstrated preliminary clinical activity and a manageable safety profile of tislelizumab combined with bevacizumab and CAPOX in patients with GEA and PD-L1 CPS <5. Larger, randomized studies are warranted to validate these findings and establish the clinical utility of this regimen.

## Ethical Approval

The study was approved by the institutional ethics committee of the Chinese People’s Liberation Army General Hospital (approval number: S2021-642-01) and was conducted in accordance with the Declaration of Helsinki and the Good Clinical Practice guidelines. The trial was registered at ClinicalTrials.gov (NCT05299476). Written informed consent was obtained from all patients prior to enrollment.

## Data Availability

The RNA sequence data used in this study were all submitted to the CNGB Sequence Archive (CNSA) of the China National GeneBank DataBase (CNGBdb), with the associated accession identifier CNP0009124.
