# Supplementary material for: First-Line Tislelizumab Combined with Bevacizumab and CAPOX for Metastatic Gastroesophageal Adenocarcinoma with Low Programmed Death-Ligand 1 Expression
Source: Cancer Commun (Lond). 2026 Apr 16;46:0024. doi: 10.34133/cancomm.0024 (PMC13084058; doi:10.34133/cancomm.0024)
Supplement: Supplementary 1 — Supplementary Methods Tables S1 to S5 Figs. S1 to S3 [file cancomm.0024.f1.docx]

**First-line Tislelizumab Combined with Bevacizumab and CAPOX for Metastatic Gastroesophageal Adenocarcinoma with Low Programmed Death-Ligand 1 Expression**

Ru Jia^1, †^, Yan-Rong Wang^1, †^, Fang-Fang Liu^1, †^, Yue Ma^1, 2, †^, Hai-Yan Si^1^, Lu Han^1^, Miao-Miao Gou^1^, Zhao-Li Tan^1^, Nan Zhang^1^, Guo-Chao Deng^1^, Meng-Jiao Fan^1^, Yue Shi^1^, Yao-Yue Zhang^1^, Yu-Shan Jia^1^, Jun-Nan Xu^3^, Xiao-Xing Su^3^, Quan-Li Han^1*^, Zhi-Kuan Wang^1, *^, Guang-Hai Dai^1, *^

^1^Senior Department of Oncology, Chinese PLA General Hospital, Beijing, P. R. China

^2^Senior Department of Oncology, Medical School of Chinese PLA, Beijing, P. R. China

^3^Kanghui Medicine, Kanghui Biotechnology Co., Ltd., Shenyang, Liaoning, P. R. China

^*^Corresponding authors

Guang-Hai Dai, Senior Department of Oncology, Chinese PLA General Hospital, Beijing, 100071, P. R. China. Email: daigh301@vip.sina.com

Zhi-Kuan Wang, Senior Department of Oncology, Chinese PLA General Hospital, Beijing, 100071, P. R. China. Email: [wangzkme@sohu.com](mailto:wangzkme@sohu.com)

Quan-Li Han, Senior Department of Oncology, Chinese PLA General Hospital, Beijing, 100071, P. R. China. Email: [hanquanli@aliyun.com](mailto:hanquanli@aliyun.com).

^†^Ru Jia^1^, Yan-Rong Wang, Fang-Fang Liu, Yue Ma contributed equally to this study.

**Supplementary Materials**

**Methods**

**Study design, procedures, endpoints, and statistical analysis**

This single-arm, single-center, open-label phase II study (NCT05299476) evaluated tislelizumab combined with bevacizumab and chemotherapy in Chinese patients with locally advanced or metastatic gastric, gastroesophageal junction, or esophageal adenocarcinoma. The study was approved by the Institutional Ethics Committee of the Chinese People's Liberation Army (PLA) General Hospital (S2021-642-01) and conducted according to the Declaration of Helsinki, Good Clinical Practice, and local regulations. All patients provided written informed consent.

Eligible patients were adults with histologically or cytologically confirmed human epidermal growth factor receptor 2 (HER2)-negative gastric, gastroesophageal junction, or esophageal adenocarcinoma, with unresectable locally advanced or metastatic disease, Eastern Cooperative Oncology Group (ECOG) performance status (PS) 0-1, programmed death-ligand 1 (PD-L1) combined positive score (CPS) <5, and no prior systemic therapy. Key exclusion criteria included uncontrolled hypertension despite standard antihypertensive therapy (systolic blood pressure ≥160 mmHg and/or diastolic blood pressure ≥100 mmHg); thromboembolic events, including stroke or transient ischemic attack, within 6 months; and conditions deemed by the investigator to confer a high risk of major bleeding, including but not limited to a history of bleeding >30 mL within the previous 3 months. In patients considered at high risk of bleeding, endoscopic evaluation could be performed, and endoscopic findings were used as the definitive assessment of bleeding risk.

Patients received tislelizumab 200 mg intravenous (IV), bevacizumab 7.5 mg/kg IV, and oxaliplatin 130 mg/m² IV on day 1 of each 3-week cycle, and capecitabine 1,000 mg/m² orally twice daily on days 1-14, for up to 8 cycles. Treatment continued until disease progression, unacceptable toxicity, withdrawal of consent, or investigator decision. Dose modification (including dose delays and/or dose reductions) were implemented for significant adverse events (AEs) and were guided by the highest (worst) toxicity grade observed. Dose adjustments were required for toxicities that did not resolve to CTCAE v5.0 Grade 0–1 or to baseline levels. Treatment was permanently discontinued if a third chemotherapy dose reduction was required or if treatment delays exceeded the prespecified 6-week/12-week time limits. Detailed procedures are provided in the study protocol. Tumor response was assessed by investigators per Response Evaluation Criteria in Solid Tumors version 1.1 (RECIST v1.1) using computed tomography (CT) or magnetic resonance imaging (MRI) at baseline and every 6 weeks for the first year, then every 9 weeks. Adverse events (AEs) were graded per Common Terminology Criteria for Adverse Events version 5.0 (CTCAE v5.0) and monitored from enrollment until 30 days after the last dose.

PD-L1 CPS analyses were performed on formalin-fixed, paraffin-embedded (FFPE) tumor tissues. PD-L1 CPS was evaluated by immunohistochemistry (IHC) using the PD-L1 IHC 22C3 pharmDx assay (Agilent Technologies, Santa Clara, CA, USA; Cat. #SK006). The CPS was calculated as the number of PD-L1-positive cells (including tumor cells, lymphocytes, and macrophages) divided by the total number of viable tumor cells, multiplied by 100. As this was a single-center study, all PD-L1 testing was conducted locally. HER2 status was determined by IHC first. Tumors with IHC scores of 0 or 1+ were classified as HER2-negative. For cases with an IHC score of 2+, fluorescence in situ hybridization (FISH) was performed; those with FISH-negative results were defined as HER2-negative. Conversely, tumors with an IHC score of 3+, or an IHC score of 2+ combined with a FISH-positive result, were defined as HER2-positive.

The primary endpoint was the 6-month progression-free survival (PFS) rate. Secondary endpoints included PFS, overall survival (OS), objective response rate (ORR), disease-control rate (DCR), duration of response (DOR), time to response (TTR), and safety.

The PFS Kaplan-Meier curves in the CheckMate 649 [1] CPS <5 subgroup were used to establish the historical benchmark. The 6-month PFS rate in this subgroup was approximately estimated to be 63% through digital extraction from the published curve using WebPlotDigitizer (https://automeris.io/v4/). Assuming a 6-month PFS rate of 84% in the present study, a sample size of 27 evaluable patients provided 80% power using a one-sided alpha level of 0.05. Considering an anticipated dropout rate of 10%, the planned sample size was 30 patients. Ultimately, 32 patients were enrolled to account for potential early withdrawal prior to the first tumor assessment and a higher-than-expected screening failure rate.

Efficacy was analyzed in the full analysis set (FAS), defined as all patients who received at least one dose of study treatment. Safety was analyzed in all patients who received at least one dose of study treatment. Continuous variables were summarized as median (range/interquartile range [IQR]) and categorical variables as frequency (%). ORR and DCR with 95% confidence intervals (CIs) were estimated using the Clopper-Pearson method. Kaplan-Meier analysis was used to estimate DOR, TTR, PFS, and OS, with 95% CIs calculated using Greenwood’s formula. Cox proportional hazards models estimated HRs and 95% CIs for factors associated with PFS and OS. Differences in biomarkers between responders (complete response [CR]/partial response [PR]) and non-responders (stable disease [SD]/progressive disease [PD]) were assessed using the Wilcoxon test. Two-sided *P*-value < 0.05 was considered significant. Analyses were performed using SPSS 25.0 and R 4.3.2.

**RNA** **extraction and sequencing**

Total RNA was extracted from frozen tumor tissues using the AlPrep DNA/RNA Mini Kit (Qiagen, Cat. #80204) and quantified with the Qubit RNA HS Assay Kit (Invitrogen, Cat. #Q32852). The RNA was then used to construct a transcriptome sequencing library with the VAHTS Universal V6 RNA-Seq Library Prep Kit for Illumina (Vazyme, Cat. #NR604-01/02). The final RNA library was quantified using the Qubit 3.0, and its fragment size was assessed with the Agilent 2100. Sequencing was performed as paired-end 150 bp on the Illumina NovaSeq 6000 platform. Raw RNA sequencing data were filtered using fastp v.0.12.6 and aligned to the human UCSC reference genome (hg19/GRCh37) with HISAT2 v.2.0.6 [2, 3]. SAMtools v0.1.19 was employed to process the alignment files for subsequent analysis [4]. Gene-level read counts for each sample were calculated using HTSeq v0.11.2 [5].

**Differentially expressed genes (DEGs) and pathway enrichment analysis**

DEGs between responders and non-responders were identified using the ‘edgeR’ R package. The DEGs that met the false discovery rate (FDR) < 0.05 and |log fold change (FC)| > 1 were selected for further exploration. Then, the gene set variation analysis (GSVA) was performed using the ‘gsva’ R package. The reference gene set ‘c2.cp.kegg.v7.5.1.symbols.gmt’ was acquired from MSigDB v7.5 (https://www.gseamsigdb.org/gsea/msigdb/index.jsp) for the GSVA analysis. Additionally, the single-sample gene set enrichment analysis (ssGSEA) score of the significantly different gene signature was compared between two subgroups.

**Fluorescent multiplex IHC staining and imaging**

Multiplex IHC staining was conducted to examine the expression of multiple biomarkers, including CD20, CD4, CD8, FOXP3, PD-1, CD68, CD86, CD163, CD56, PD-L1, CD31, CD34, SMA, FAP, CD66B, and pan-cytokeratin (pan-CK), in tumor tissues using three antibody panels (**Supplementary Table S5**). Tissue samples were obtained within 30 minutes of surgical resection or biopsy and subsequently fixed in formalin for 24-48 hours. Following standard protocols, the specimens underwent dehydration and paraffin embedding. For analysis, four sequential sections (4 µm thick) were prepared from each paraffin block. One section was reserved for hematoxylin and eosin (H&E) staining, while the remaining three FFPE slides were incubated at 60°C for 12 hours to remove paraffin. Deparaffinization was performed using xylene, followed by rehydration in a graded alcohol series. Antigen retrieval was carried out via microwave heating in EDTA buffer (pH 9.0). To minimize nonspecific binding, tissue sections were treated with a commercial blocking solution (Panovue, Cat. #10018001120) for 10 minutes.

The staining protocol involved sequential incubation with primary antibodies and horseradish peroxidase-conjugated secondary antibodies, followed by tyramide signal amplification (TSA). After each TSA cycle, slides underwent heat-mediated antigen retrieval and antibody stripping to enable subsequent staining rounds. Finally, nuclei were counterstained with 4’, 6-diamidino-2-phenylindole (DAPI).

The interleukin-17A (IL-17A) IHC staining was semi-quantitatively evaluated using the histological score (H-score) criteria as follows: Positive expression was defined as cytoplasmic staining appearing light yellow to brown. Staining intensity was scored as 0 (negative, no staining or similar to background), 1 (weak, light yellow or only a few cells with yellow-brown staining), 2 (moderate, intermediate intensity), or 3 (strong, most cells displaying distinct yellow-brown staining). The percentage of positively stained cells was scored as 0 (<5%), 1 (5%-25%), 2 (25%-50%), 3 (50%-75%), or 4 (≥75%) (**Supplementary Table S5**). The final score was calculated by multiplying the staining intensity score by the positive cell percentage score.

**Chemiluminescent immunoassay**

Serum IL-17A levels were quantified using a chemiluminescent immunoassay based on an automated chemiluminescence analyzer (Guangzhou Kangrun Biotechnology Co., Ltd., China). The assay utilized paramagnetic microparticles coated with capture antibodies and acridinium ester-labeled detection antibodies. After incubation, unbound components were removed via magnetic separation, and chemiluminescent signals were quantified post-substrate addition, with signal intensity directly proportional to IL-17A levels. Samples exceeding the linear range were automatically diluted 1:10 by the instrument.

**Reference**

1. Zhao JJ, Yap DWT, Chan YH, Tan BKJ, Teo CB, Syn NL, et al. Low Programmed Death-Ligand 1-Expressing Subgroup Outcomes of First-Line Immune Checkpoint Inhibitors in Gastric or Esophageal Adenocarcinoma. J Clin Oncol. 2022;40(4):392-402.

2. Chen S, Zhou Y, Chen Y, Gu J. fastp: an ultra-fast all-in-one FASTQ preprocessor. Bioinformatics. 2018;34(17):i884-i90.

3. Kim D, Langmead B, Salzberg SL. HISAT: a fast spliced aligner with low memory requirements. Nat Methods. 2015;12(4):357-60.

4. Li H, Handsaker B, Wysoker A, Fennell T, Ruan J, Homer N, et al. The Sequence Alignment/Map format and SAMtools. Bioinformatics. 2009;25(16):2078-9.

5. Putri GH, Anders S, Pyl PT, Pimanda JE, Zanini F. Analysing high-throughput sequencing data in Python with HTSeq 2.0. Bioinformatics. 2022;38(10):2943-5.

**Supplementary Table S1. Baseline characteristics**

| **Characteristics** | **FAS (*n* = 32)** |
| --- | --- |
| Age, median (range), years | 59 (27-72) |
| Male, *n* (%) | 22 (68.8) |
| ECOG performance status, *n* (%) | |
| 0 | 12 (37.5) |
| 1 | 20 (62.5) |
| Primary tumor location at diagnosis, *n* (%) | |
| Gastroesophageal junction | 8 (25.0) |
| Gastric | 24 (75.0) |
| Prior primary tumor resection, *n* (%) | 6 (18.8) |
| Differentiation type, *n* (%) | |
| Low | 30 (93.8) |
| Intermediate | 2 (6.2) |
| Number of metastatic sites, *n* (%) | |
| ≥2 | 23 (71.9) |
| 1 | 9 (28.1) |
| Site of metastases, *n* (%) | |
| Lymph node | 30 (93.8) |
| Peritoneum | 14 (43.8) |
| Liver | 10 (31.2) |
| Lung | 3 (9.4) |
| HER2 expression, *n* (%) | |
| 0 | 17 (53.1) |
| 1+ | 10 (31.3) |
| 2+ | 5 (15.6) |
| PD-L1 CPS, *n* (%) | |
| <1 | 11 (34.4) |
| 1-4 | 21 (65.6) |
| Baseline CEA, ng/mL | |
| Median (IQR) | 1.9 (0.9-4.6) |
| Baseline CA19-9, U/mL |  |
| Median (IQR) | 11.6 (5.6-29.4) |
| Baseline CA72-4, U/mL | |
| Median (IQR) | 7.3 (1.7-24.3) |

Abbreviations: FAS, full analysis set; ECOG, Eastern Cooperative Oncology Group; HER2, human epidermal growth factor receptor 2; CPS, combined positive score; CEA, carcinoembryonic antigen; IQR, interquartile range; CA, cancer antigen; PD-L1, programmed cell death protein 1.

**Supplementary Table S2. Tumor responses**

| Tumor response | Full analysis set  (*n* = 32) | Patients with baseline measurable disease  (*n* = 27) |
| --- | --- | --- |
| Best response, *n* (%) | | |
| Complete response | 0 (0.0) | 0 (0.0) |
| Partial response | 18 (56.2) | 18 (66.7) |
| Stable disease | 14 (43.8)^a^ | 9 (33.3) |
| Progressive disease | 0 (0.0) | 0 (0.0) |
| Objective response rate, *n* (%, 95% CI) | 18 (56.2, 37.7-73.6) | 18 (66.7, 46.0-83.5) |
| Disease control rate, *n* (%, 95% CI) | 32 (100.0, 89.1-100.0) | 27 (100.0, 87.2-100.0) |
| Duration of response, median (95% CI), months | 8.8 (6.2-11.4) | 8.8 (6.2-11.4) |
| Time to response, median (95% CI), months | 2.6 (0.5-4.7) | 1.7 (1.1-2.2) |

^a^ Including 5 patients with non-target lesions at baseline. Abbreviations: CI, confidence interval.

**Supplementary Table S3. Subsequent anti-cancer therapy**

| Subsequent anti-cancer therapy | Patients (*n* = 20)^a^ |
| --- | --- |
| Chemotherapy | 17 (85.0%) |
| Nab-paclitaxel | 11 (55.0%) |
| CAPOX | 4 (20.0%) |
| Irinotecan-based regimen | 4 (20.0%) |
| Disitamab vedotin | 2 (10.0%) |
| Anti-angiogenesis therapy^b^ | 12 (60.0%) |
| PD-1 antibody | 10 (50.0%) |
| Surgery | 3 (15.0%) |
| Intraperitoneal chemotherapy | 3 (15.0%) |
| Radiotherapy | 3 (15.0%) |

^a^Among 23 patients who discontinued the study, 20 patients received subsequent anti-cancer therapy. ^b^ Anti-angiogenic therapies administered in this study included bevacizumab and apatinib. Abbreviations: CAPOX, capecitabine plus oxaliplatin; PD-1, programmed cell death protein 1; Nab-paclitaxel, nanoparticle albumin-bound paclitaxel.

**Supplementary Table S4. Treatment-related adverse events with an incidence ≥5% in the safety set (*n* = 32)**

| **Treatment-related adverse events** | **Any**  **grade** | **Grade 1** | **Grade 2** | **Grade 3** | **Grade 4** |
| --- | --- | --- | --- | --- | --- |
| Decreased neutrophil count | 19 (59.4) | 6 (18.8) | 6 (18.8) | 7 (21.9) | 0 (0.0) |
| Nausea | 15 (46.9) | 8 (25.0) | 7 (21.9) | 0 (0.0) | 0 (0.0) |
| Decreased platelet count | 13 (40.6) | 3 (9.4) | 10 (31.3) | 0 (0.0) | 0 (0.0) |
| Anorexia | 13 (40.6) | 10 (31.3) | 3 (9.4) | 0 (0.0) | 0 (0.0) |
| Fatigue | 10 (31.3) | 9 (28.1) | 1 (3.1) | 0 (0.0) | 0 (0.0) |
| Vomiting | 9 (28.1) | 6 (18.8) | 3 (9.4) | 0 (0.0) | 0 (0.0) |
| Hand-foot syndrome | 9 (28.1) | 4 (12.5) | 2 (6.3) | 3 (9.4) | 0 (0.0) |
| Anemia | 9 (28.1) | 4 (12.5) | 4 (12.5) | 1 (3.1) | 0 (0.0) |
| Increased ALT/AST | 7 (21.9) | 4 (12.5) | 3 (9.4) | 0 (0.0) | 0 (0.0) |
| Rash | 6 (18.8) | 1 (3.1) | 4 (12.5) | 1 (3.1) | 0 (0.0) |
| Peripheral neuropathy | 5 (15.6) | 5 (15.6) | 0 (0.0) | 0 (0.0) | 0 (0.0) |
| Diarrhea | 4 (12.5) | 2 (6.3) | 1 (3.1) | 1 (3.1) | 0 (0.0) |
| Mucositis^a^ | 4 (12.5) | 0 (0.0) | 2 (6.3) | 2 (6.3) | 0 (0.0) |
| Increased blood bilirubin | 3 (9.4) | 1 (3.1) | 0 (0.0) | 1 (3.1) | 1 (3.1) |
| Hypothyroidism | 3 (9.4) | 0 (0.0) | 3 (9.4) | 0 (0.0) | 0 (0.0) |
| Bleeding^b^ | 3 (9.4) | 0 (0.0) | 3 (9.4) | 0 (0.0) | 0 (0.0) |
| Hypertension | 2 (6.3) | 0 (0.0) | 1 (3.1) | 1 (3.1) | 0 (0.0) |

Data are shown as *n* (%).

^a^ Mucositis included 2 patients with grade 2 oral ulcer, 1 with grade 3 cheilitis, and 1 with grade 3 esophagitis.

^b^ Grade 2 bleeding included 1 with upper gastrointestinal bleeding, 1 with cheilitis-related bleeding and 1 with gingival bleeding. Abbreviations: ALT, alanine aminotransferase; AST, aspartate aminotransferase.

**Supplementary Table 5. Antibodies used in the study.**

| **Marker** | **Catalog number** | **Application** | **Dilution** | **Manufacturer** |
| --- | --- | --- | --- | --- |
| CD20 | ab78237 | AB_1640323 | 1:200 | Abcam |
| CD4 | ZM0418 | AB_2890106 | 1:400 | Zsbio |
| CD8A | CST70306 | AB_2799781 | 1:200 | CST |
| FOXP3 | BX50188 | AB_3738531 | 1:200 | Biolynx |
| PD-1 | CST86163 | AB_2728833 | 1:200 | CST |
| CD56 | CST3576 | AB_2149540 | 1:200 | CST |
| CD68 | CST76437 | AB_2799882 | 1:400 | CST |
| CD86 | CST91882 | AB_2797422 | 1:200 | CST |
| CD163 | CST93498 | AB_2800204 | 1:300 | CST |
| PD-L1 | CST13684 | AB_2687655 | 1:200 | CST |
| CD66b | GTX19779 | AB_423727 | 1:600 | Genentech |
| CD31 | CST3528 | AB_2160882 | 1:800 | CST |
| CD34 | CST3569 | AB_2074374 | 1:400 | CST |
| FAP-α | Ab207178 | AB_2864720 | 1:200 | Abcam |
| α-SMA | Ab5694 | AB_2223021 | 1:500 | Abcam |
| PanCK | C2562 | AB_476839 | 1:1000 | Sigma |
| IL-17A | DF6127 | AB_2838094 | 1:200 | Affinity |

Abbreviations: CD20, cluster of differentiation 20; FOXP3, forkhead box P3; PD-1, programmed cell death protein 1; PD-L1, programmed death-ligand 1; FAP-α, fibroblast activation protein alpha; α-SMA, alpha-smooth muscle actin; PanCK, pan-cytokeratin; IL-17A, interleukin-17A.


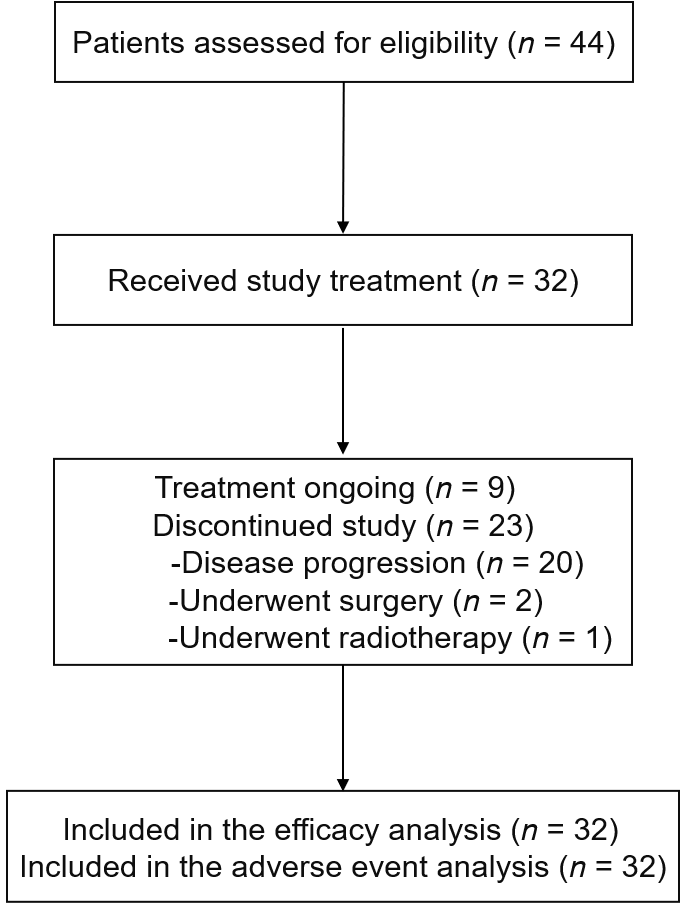


**Supplementary Figure S1. CONSORT‑style flow diagram of patient screening, enrollment, treatment, and analysis**

The diagram illustrates the disposition of patients from initial screening through study treatment and analysis in the trial evaluating tislelizumab combined with bevacizumab and CAPOX as first‑line therapy for HER2‑negative, PD‑L1 CPS <5 locally advanced or metastatic GEA. A total of 44 patients were screened for eligibility. Of these, 12 were excluded: 8 due to PD‑L1 CPS ≥5, 2 due to HER2‑positive status, and 2 due to endoscopically assessed active bleeding. The remaining 32 patients received the assigned treatment and were included in both the efficacy and safety analysis sets. As of the data cutoff, 9 patients remained on treatment, while 23 had discontinued treatment due to disease progression (*n* = 20), surgical intervention (*n* = 2), or radiotherapy (*n* = 1). Abbreviations: CAPOX, capecitabine plus oxaliplatin; CPS, combined positive score; HER2, human epidermal growth factor receptor 2; PD‑L1, programmed death‑ligand 1; GEA, gastroesophageal adenocarcinoma.

**
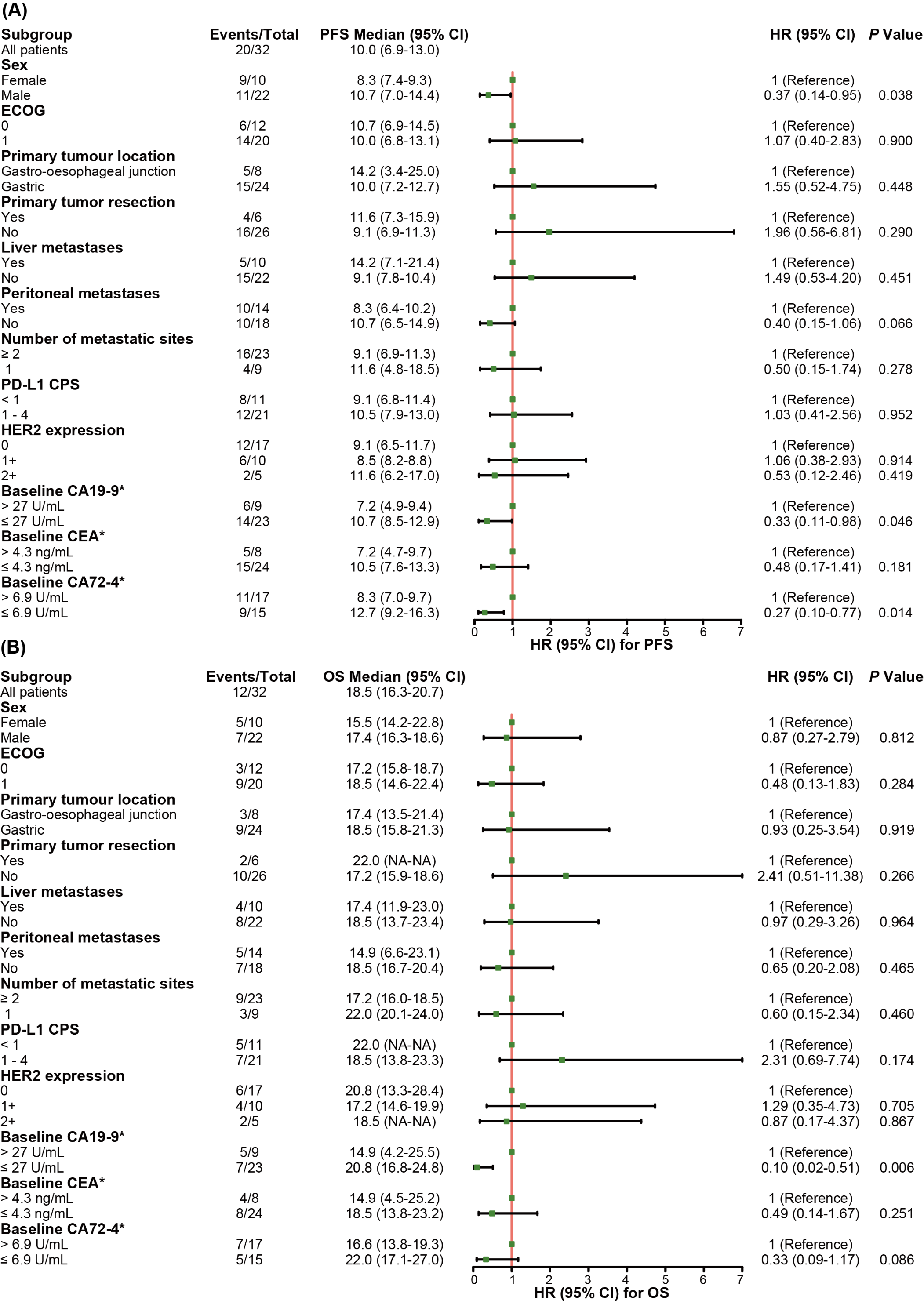
**

**Supplementary Figure S2. Forest plots of PFS and OS across patient subgroups**

**(A)** Forest plot of HRs (95% CI) for PFS in the full analysis set (*n* = 32), stratified by baseline clinical and pathological characteristics. Median PFS was 10.0 (95% CI 6.9-13.0) months. Each row shows events/total, median PFS (95% CI), and HR (95% CI). The vertical dashed line indicates HR = 1.0. **(B)** Forest plot of HRs (95% CI) for OS in the same subgroups. Median OS was 18.5 (95% CI 16.3-20.7) months. Each row shows events/patients, median OS (95% CI), and HR (95% CI). The red vertical line indicates HR = 1.0. ^*^Biomarker subgroups were defined using normal cutoff values at our center. Analyses were performed with Cox proportional hazards models. Abbreviations: CA, carbohydrate antigen; CEA, carcinoembryonic antigen; CI, confidence interval; CPS, combined positive score; ECOG, Eastern Cooperative Oncology Group performance status; HER2, human epidermal growth factor receptor 2; HR, hazard ratio; NA, not available; OS, overall survival; PD-L1, programmed death ligand 1; PFS, progression-free survival.

**
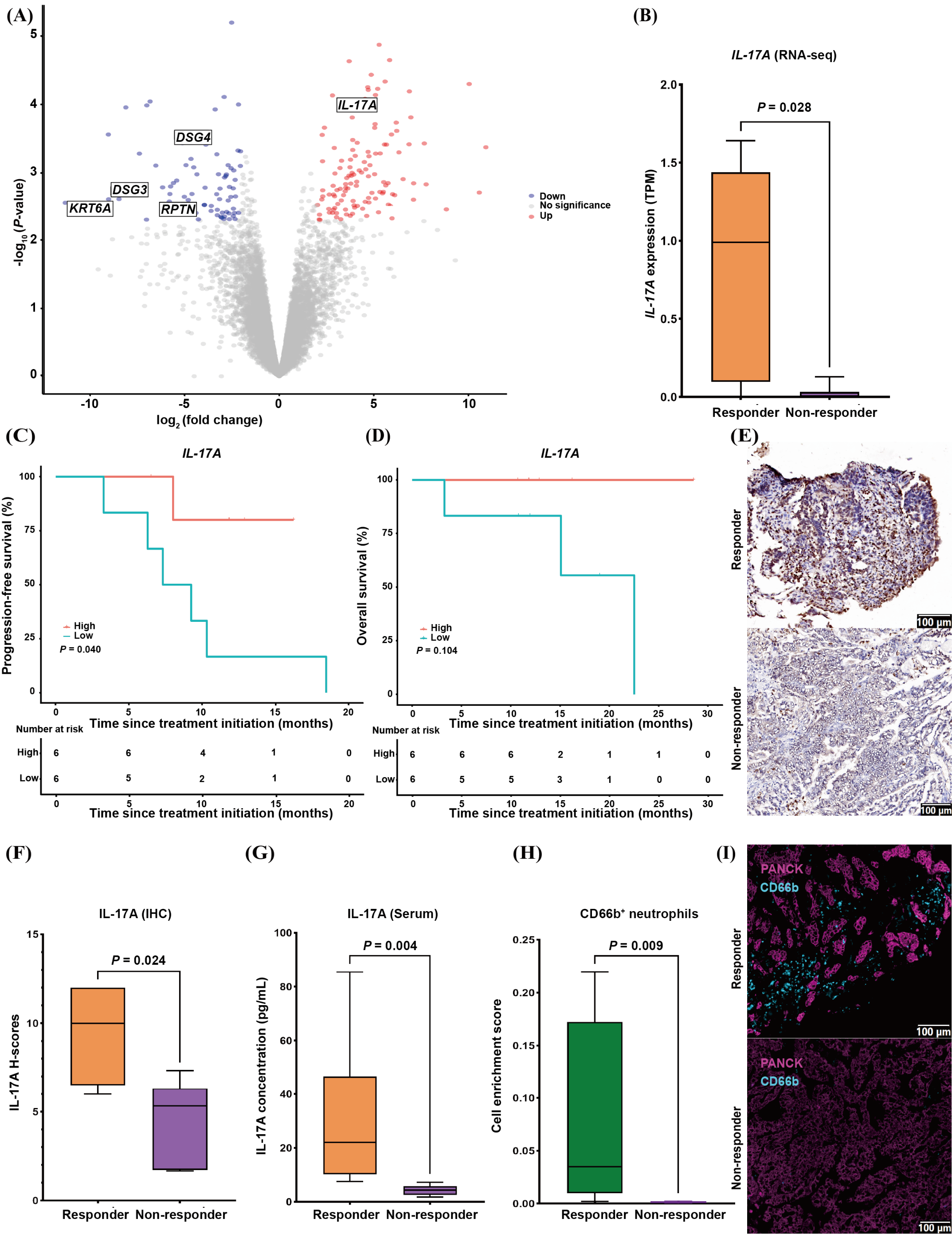
**

**Supplementary Figure S3. Exploratory analyses of molecular and immune correlates of tumor response in patients with available tumor tissue.**

Exploratory analyses were conducted in 12 patients with tumor tissue (responders: PR, *n* = 6; non-responders: SD, *n* = 6). **(A)** Volcano plot of differentially expressed genes (DEGs) between responders and non-responders based on tumor RNA-seq. The y-axis shows −log₁₀ (*P*-value), and the x-axis shows log₂ (fold change). Significantly dysregulated genes, defined by the thresholds of *P*-value < 0.005 and |log₂FC| > 1, are colored: blue for genes upregulated in non-responders (representative genes, e.g., *KRT6A*, *DSG3*, *DSG4*, and *RPTN* are labeled) and red for genes upregulated in responders. **(B)** Box plot of *IL-17A* (RNA-seq) expression (TPM) in responders vs. non-responders (Mann-Whitney U test, *P* = 0.028). **(C, D)** Kaplan-Meier curves for PFS **(C)** and OS **(D)** stratified by median *IL-17A* expression (log-rank test). **(E)** Representative IHC images of IL-17A protein expression in one responder (top) and one non-responder (bottom). **(F)** Box plot of IL-17A H-scores in responders vs. non-responders (*n* = 12; Mann-Whitney U test). **(G)** Box plot of baseline serum IL-17A levels by ELISA in responders vs*.* non-responders (*n* = 11; Mann-Whitney U test). **(H)** Bar plot of CD66b^+^ neutrophils intensity ratio from mIHC in responders vs. non-responders (*n* = 12; Mann-Whitney U test). **(I)** Representative mIHC images of CD66b⁺ neutrophils (green) and PANCK (red) from one responder and one non-responder. **Abbreviations:** CD66b, granulocyte marker; CI, confidence interval; DAPI, 4’, 6-diamidino-2-phenylindole; DEG, differentially expressed gene; DSG, desmoglein; ELISA, enzyme-linked immunosorbent assay; GSVA, gene set variation analysis; H-score, histochemical score; IHC, immunohistochemistry; IL-17A, interleukin-17A; KRT6A, keratin-6A; mIHC, multiplex IHC; OS, overall survival; PANCK, pan-cytokeratin; PFS, progression-free survival; PR, partial response; RPTN, repetin; RNA-seq, RNA sequencing; SD, stable disease; ssGSEA, single-sample gene set enrichment analysis; TPM, transcripts per million.
